# Enhanced Magnetoelectric Coupling in BaTiO_3_-BiFeO_3_ Multilayers—An Interface Effect

**DOI:** 10.3390/ma13010197

**Published:** 2020-01-02

**Authors:** Stefan Hohenberger, Johanna K. Jochum, Margriet J. Van Bael, Kristiaan Temst, Christian Patzig, Thomas Höche, Marius Grundmann, Michael Lorenz

**Affiliations:** 1 Felix-Bloch-Institut für Festkörperphysik, Universität Leipzig, Linnéstraße 5, D-04103 Leipzig, Germanymlorenz@physik.uni-leipzig.de (M.L.); 2 Quantum Solid State Physics, Celestijnenlaan 200D, B-3001 Leuven, Belgium; 3 Heinz Maier-Leibnitz Zentrum, Lichtenbergstr. 1, D-85747 Garching, Germany; 4 Center for Applied Microstructure Diagnostics, Fraunhofer-Institut für Mikrostruktur von Werkstoffen und Systemen, Walter-Hülse-Straße 1, D-06120 Halle, Germany

**Keywords:** multiferroic, magnetoelectric, oxide superlattices

## Abstract

Combining various (multi-)ferroic materials into heterostructures is a promising route to enhance their inherent properties, such as the magnetoelectric coupling in BiFeO_3_ thin films. We have previously reported on the up-to-tenfold increase of the magnetoelectric voltage coefficient $\alpha_{\mathrm{ME}}$ in BaTiO_3_-BiFeO_3_ multilayers relative to BiFeO_3_ single layers. Unraveling the origin and mechanism of this enhanced effect is a prerequisite to designing new materials for the application of magnetoelectric devices. By careful variations in the multilayer design we now present an evaluation of the influences of the BaTiO_3_-BiFeO_3_ thickness ratio, oxygen pressure during deposition, and double layer thickness. Our findings suggest an interface driven effect at the core of the magnetoelectric coupling effect in our multilayers superimposed on the inherent magnetoelectric coupling of BiFeO_3_ thin films, which leads to a giant $\alpha_{\mathrm{ME}}$ coefficient of 480 Vcm${}^{-1}$ Oe${}^{-1}$ for a 16×(BaTiO_3_-BiFeO_3_) superlattice with a 4.8 nm double layer periodicity.

## 1. Introduction

The control of magnetism by electric fields and vice versa, the control of ferroelectric polarization by magnetic fields, in magnetoelectric (ME) multiferroics promises great advantages in realizing a number of novel applications. Since their discovery, they have been successfully implemented in the field of spintronics [1]. In the field of sensor devices, a typical route to reach strong effective ME coupling effects is the operation at mechanical resonance frequencies [2]. However en route towards stable ME memory devices and other low-frequency out-of-resonance applications, other means have to be explored to enhance the typically weak coupling effect. Artificial multiferroic heterostructures [3] and composite materials [4] show great promise in this respect and offer a way to circumnavigate the problem of the scarcity of naturally occurring single phase multiferroics [5].

Apart from generating new artificial multiferroic heterostructures from purely ferroelectric and ferromagnetic constituent compounds, composites can also be used to enhance the properties of an intrinsic multiferroic. In 2014 we first reported on the enhanced ME coupling in thin film BaTiO_3_-BiFeO_3_ (BTO-BFO) composites with 2:1 and 1:2 composition ratios and 15×(BaTiO_3_ (BTO)-BiFeO_3_ (BFO)) multilayers [6]. The intrinsic multiferroic BFO and the ferroelectric BTO both possess perovskitic unit cells with closely matched lattice constants [6]. We measured an enhanced magnetoelectric voltage coefficient ($\alpha_{\mathrm{ME}}$) of 21 Vcm${}^{-1}$Oe${}^{-1}$ for a composite film with a 2:1 BTO-BFO composition ratio relative to the 4.2 Vcm${}^{-1}$Oe${}^{-1}$ measured for a BFO single layer. Further experiments showed that, while the enhanced ME effect was larger in composites than in multilayers, it proved far more malleable in multilayers. We also reported for the first time the characteristic dependencies of $\alpha_{\mathrm{ME}}$ on an external DC bias magnetic field $H_{\mathrm{bias}}$ for these composites and multilayers [6]. While the BFO single layers and composite films show a maximum and subsequent decrease in $\alpha_{\mathrm{ME}}$ when $H_{\mathrm{bias}}$ is increased from 0 T to 6 T, similar to the behavior of bulk samples [7], multilayers show a saturating behavior [6]. This field dependency was observed for all BTO-BFO multilayer samples since [8,9,10,11,12,13]. By variation of the $p_{O_{2}}$ pressure during growth we found an increase of oxygen octahedral tilt [8] and micro-strain [9] with lowered $p_{O_{2}}$ that correlated with a decrease of the respective $\alpha_{\mathrm{ME}}$ values. Note, however, that the pulse numbers were kept constant for these experiments, yielding increasingly larger double layer thickness ($d_{\mathrm{dl}}$) values with decreasing $p_{O_{2}}$. A decrease of the BFO sublayer thickness with constant BTO thickness leads to a significant increase of the measured $\alpha_{\mathrm{ME}}$ value [12,13,14]. Jochum et al. showed that lowering $d_{\mathrm{BFO}}$ from 50 nm to 5 nm led to an increase of $\alpha_{\mathrm{ME}}$ from 11 Vcm${}^{-1}$Oe${}^{-1}$ to 56 Vcm${}^{-1}$Oe${}^{-1}$, which was accompanied by an increasing asymmetry of the hyperfine field distribution [12]. Simultaneously the temperature dependence of $\alpha_{\mathrm{ME}}$ changed from monotonically falling to monotonically rising, indicating a change of the dominant coupling mechanism [12]. The variation of the volume fraction of the ferroelectric and magnetostrictive phases in artificial magnetoelectric multiferroic composites is expected to have a strong influence on the magnetoelectric coupling in purely strain-mediated heterostructures according to theoretic calculations [15,16]. This has often been confirmed in experiments; for reviews detailing such examples, we refer to [4,17]. It is debatable, however, whether this theory should be applicable in this case, as (a) the combination BTO-BFO is ferroelectric–multiferroic rather than just ferroelectric–ferromagnetic (and strictly speaking bulk BFO is anti-ferromagnetic, not ferromagnetic); (b) the BTO-BFO ratio variation based on BFO thickness variation leads to an overall thickness variation; and (c) it is not entirely clear, if a purely strain-mediated coupling effect lies at the core of the observed enhanced ME coupling. Through Mössbauer spectroscopy, we found a tilt of the preferential magnetic orientation from in-plane for single layer BFO films to out-of-plane for multilayers [10]. The number of double layer repetitions in a sample enhances this effect and also leads to an increase of $\alpha_{\mathrm{ME}}$ [11].

In summary, our previous specific choices in design and deposition parameters give rise to some additional questions concerning their influence on the ME coupling in BTO-BFO multilayers:

- Does the BTO-BFO thickness ratio have an explicit influence on $\alpha_{\mathrm{ME}}$?
- Does an explicit dependence on the double layer thickness $d_{\mathrm{dl}}$ exist?
- Can the $p_{O_{2}}$ dependence be verified with constant $d_{\mathrm{dl}}$?

We have designed a number of sample series with close control of the BTO-BFO thickness ratio and double layer thickness in order to answer these questions. In the following, we present the details of the sample preparation and characterization in Section 2. In Section 3 and Section 4 we present and discuss the results of our structural, electric, magnetic, and magnetoelectric measurements, respectively.

## 2. Materials and Methods

### 2.1. Sample Preparation

Samples were prepared by pulsed laser deposition (PLD) using a 248 nm Coherent LPX PRO 305 F KrF excimer laser (Coherent, Dieburg, Germany). At 650 mJ per pulse, the energy density at the target was set to 2.0 J/cm${}^{2}$, with a target-to-substrate distance of 10 cm. Thin films were deposited simultaneously onto four 5 mm×5 mm etched and annealed SrTiO_3_ (STO) and STO:Nb (001) substrates placed in a rotating sample holder. The center of the sample holder was horizontally offset by 3 cm relative to the plasma plume position to ensure lateral homogeneity. A resistive heater was used, for details see [18]. Pulse numbers were determined by depositing single layers and measuring their thicknesses by X-ray reflectometry (XRR), in order to reach the targeted thicknesses. The ceramic targets were prepared from 5N BaTiO_3_, and 5N Bi_2_O_3_ and 5N Fe_2_O_3_ powders by ball-milling and sintering in air at 1300 ${}^{\circ }C$ for 6 h or 800 ${}^{\circ }C$ for 12 h, respectively. ∼100 nm Pt top contacts were deposited by DC magnetron sputtering in 0.025 m Ar at room temperature.

The standard sample design, based upon which deposition parameters were systematically varied was as follows: 16 double-layers with $d_{\mathrm{dl}}$ of 20 nm, identical $d_{\mathrm{dl}}$ of BTO and BFO, deposited in 0.25 m at ∼690 ${}^{\circ }C$, starting with a BTO and ending on a BFO layer. An initial 3 nm seed layer of BTO was deposited at a laser repetition rate of 1 Hz, after which the frequency was increased to 15 Hz.Three groups of samples were designed to test the influence of (a) the BTO-BFO thickness ratio (R*XX*); (b) O_2_ pressure (P*YY*); and (c) double layer thickness (D*ZZZ*). Table 1 gives an overview over the samples used in this study and explains the naming scheme. Sample P25 is the ’standard’ sample with a BTO-BFO thickness ratio of 1:1, deposited in 0.25 mbar O_2_, and with a nominal thickness of 20 nm and hence supplements the thickness ratio series as R05 and the thickness series as D200. The overall $d_{\mathrm{dl}}$ was maintained constant at ∼20 nm for series (a) and (b) to exclude the influence of $d_{\mathrm{dl}}$, whereas for series (c) $d_{\mathrm{dl}}$ was explicitly varied with a constant BTO-BFO 1:1 thickness ratio. The thinnest sample D48 consists of only 6 unit cells of each BaTiO_3_ (BTO) and BiFeO_3_ (BFO) per double layer.

### 2.2. Structural Characterization

A PANalytical X’pert MRD PRO diffractometer (Malvern Panalytical, Almelo, The Netherlands) with Cu K${}_{\alpha }$ radiation using a parabolic mirror and PIXcel${}^{3D}$ detector was used to measure X-ray diffraction (XRD) 2θ-ω scans and reciprocal space map (RSMs). Lattice parameters were calculated by using the substrate peak positions as internal standard. A proportional detector in combination with a parallel plate collimator was used to record XRR scans. Transmission electron microscope (TEM) experiments were performed in a FEI TITAN${}^{3}$ G2 80–300 microscope (FEI Europe Nano Port, Eindhoven, The Netherlands) at 300 keV acceleration voltage. Cross sections were prepared by wedge-polishing and subsequent ion milling along the (110) azimuth of the substrate.

### 2.3. Magnetic, Ferroelectric and Magnetoelectric Characterization

Ferroelectric hysteresis measurements were carried out on the STO:Nb-multilayer-Pt capacitors using a TF 2000 HS model thin film analyzer (aixACCT, Aachen, Germany). Electric field *E* dependent current *I* and polarization *P* curves were recorded. All measurements were carried out at 1 kHz in the dynamic hysteresis mode with triangular excitation pulses. A description of the measurement procedure of the dynamic hysteresis measurement can be found in Figure S1. Magnetic measurements were performed using a Quantum Design physical property measurement system (PPMS) (Quantum Design, Darmstadt, Germany) equipped with a vibrating sample magnetometer (VSM). The out-of-plane longitudinal magnetoelectric voltage coefficient $\alpha_{ME,\;33}=\frac{dE}{dH}=\frac{U_{\mathrm{ME}}}{d_{\mathrm{tot}}}=\alpha_{\mathrm{ME}}$ was measured in another quantum design PPMS with a Stanford Research SR830 lock-in amplifier (Stanford Research Systems, Inc., Sunnyvale, CA, USA) at KU Leuven. The voltage response $U_{\mathrm{ME}}$ to a 10 Oe AC magnetic field was measured across a capacitor structure at a frequency of 1 kHz. For more details on this method, see [6,7]. If not stated otherwise, $\alpha_{\mathrm{ME}}$ refers to values measured in 0 T bias field at 300 K in the following. Test measurements performed on ∼300 nmBaTiO_3_ and BiFeO_3_ films gave $\alpha_{\mathrm{ME}}$ values of 0.01 Vcm${}^{-1}$Oe${}^{-1}$ and 6.42 Vcm${}^{-1}$Oe${}^{-1}$, respectively. The value for BTO represents the noise level of this measurement setup, the value for BFO is close to other literature values [19].

## 3. Results

### 3.1. X-ray Diffraction Measurements

**Figure 1:**
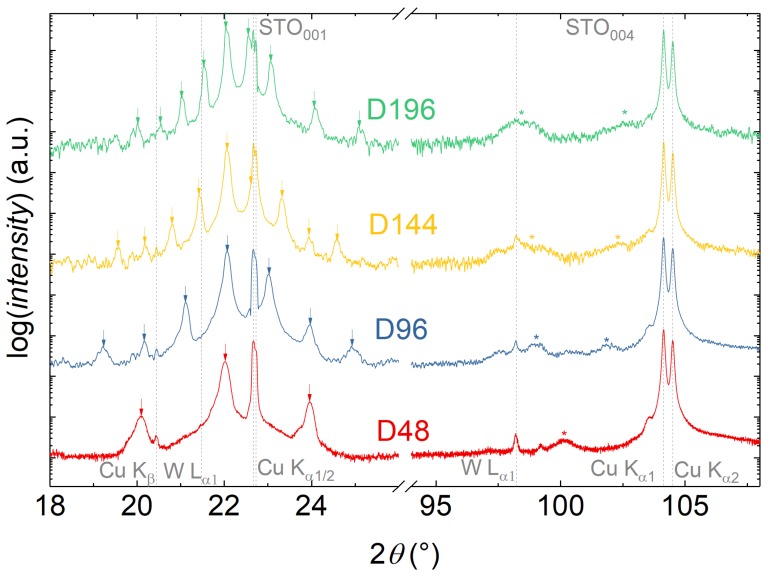
Symmetric 2θ-ω (00l) XRD scans for the samples of the $d_{\mathrm{dl}}$-series (D48-D192). The gray dashed lines indicate the positions of the substrate STO (00l) reflexes with Cu $K_{\alpha 1/2}$ splitting, Cu $K_{\beta }$, and W $L_{\alpha }$ spectral line contributions as marked, the arrows indicate the positions of superlattice fringe peaks, and the asterisks the (004) superstructure peaks. The measurement on sample D48 was performed with a smaller step size and a four times longer integration time to improve the signal to noise ratio.

In X-ray 2θ-ω scans we could confirm the high quality of the produced multilayers. Figure 1 shows the 2θ-ω measurements for the samples of the thickness series, additionally the scans for the $p_{O_{2}}$ and BTO-BFO thickness ratio series can be found in Figure S2. Multilayer fringe peaks are clearly visible for all samples except the one grown at 0.01 mbar and allow the calculation of the $d_{\mathrm{dl}}$ values tabulated in Table 2. For all samples deposited in 0.25 mbar with ∼20 nm $d_{\mathrm{dl}}$ the main superstructure peak is more intense than the substrate peak and fringe peaks are visible up to the seventh order. In case of the high BTO-content samples, fringe peaks are even visible at high angles superimposed on the (004) film peaks (see Figure S1b). Interestingly, within the $p_{O_{2}}$ series, both the 0.25 mbar (P25) and 0.05 mbar (P05) samples show well defined multilayer fringe peaks indicative of a high degree of order and low interface roughness, but the sample grown at intermediary 0.10 mbar (P10) shows peak broadening almost to the same extent as the low pressure 0.01 mbar (P01) sample (see Figure S2a).

**Figure 2:**
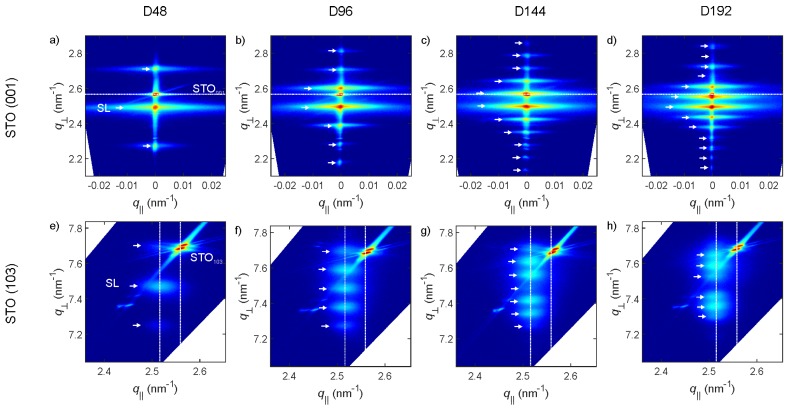
Reciprocal space map (RSM) around the STO 001 (**a**–**d**) and 103 (**e**–**h**) peaks for samples D48 (**a**,**e**), P96 (**b**,**f**), D144 (**c**,**g**), and D192 (**d**,**i**). The white arrows mark the positions of superlattice peaks, the horizontal line in (**a**–**d**) marks the out-of-plane position of the STO (001) peak, the vertical lines in (**e**–**g**) mark the in-plane position of the superlattice and STO (103) peaks, respectively, as labeled in (**e**).

Reciprocal space maps around the STO (001) and skew-asymmetric (103) peaks, exemplarily depicted for the $d_{\mathrm{dl}}$ series in Figure 2 give an even deeper insight into the epitaxial quality of the multilayer samples. Figures S3 and S4 additionally show the RSMs for the ratio and $p_{O_{2}}$ series, respectively. The full width at half maximum (FWHM) of the main layer peak in the RSM around the (001) STO peak can be used as a gauge of the samples’ mosaicity and is tabulated in Table 2. As a reference, the FWHM of the (001) STO substrate peak is typically 0.02°. The FWHM of the $p_{O_{2}}$ series correlates well with the washing out of the superlattice peaks, as for P25 and P05 values below 0.09° are obtained, while for the P01, which shows no multilayer fringe peaks, the value is as high as 0.46°. In case of the BTO-BFO thickness ratio series, an excessive proportion of BFO content (R01, BTO thickness only 2.1 nm) leads to an increased mosaicity (0.21°), while the optimum of 0.04° is reached for sample R07 with 13.4 nm and 6.0 nm BTO and BFO thickness, respectively. All samples of the $d_{\mathrm{dl}}$ series possess very low mosaicity with FWHMs around 0.09°, except the thinnest sample D48, for which an even lower value of 0.05° is obtained.

According to the lack of $q_{\|}$ alignment of substrate and film peaks in asymmetric (103) RSM measurements, the multilayers grow relaxed with respect to the substrate, but the individual layers are coherently strained to each other (see Figure 2e–f and [9,14]). The average in-plane lattice constants of the multilayers $a_{\|\mathrm{ave}}$, as extracted from (103) RSMs are tabulated in Table 2, and additionally illustrated in Figure S5. Similarly to the trend reported in [13], the thickness ratio of BTO to BFO leads to a modulation of $a_{\|\mathrm{ave}}$. For low BTO-content (R01) $a_{\|\mathrm{ave}}$ is close to the bulk $a_{p.c.}$ value of BFO and close to the bulk value for *a* of BTO for high BTO-content (R09). The oxygen pressure during deposition does not lead to a linear change of $a_{\|\mathrm{ave}}$, but rather the largest value is measured for the 0.10 mbar sample and declines for both larger and lower $p_{O_{2}}$. The change of $d_{\mathrm{dl}}$ with constant BTO-BFO thickness ratio does not lead to any significant changes of $a_{\|\mathrm{ave}}$.

Additionally, we were able to extract approximate individual layer thicknesses by fitting XRR scans (not shown) for most samples, the resulting values are listed in Table 2. While the individual BTO and BFO thicknesses vary slightly from the target values, the overall $d_{\mathrm{dl}}$ is mostly maintained close to the 20 nm standardized target value for the thickness ratio and $p_{O_{2}}$ series. For the ratio series, the actual BTO thickness fraction ranges from ∼0.14 to ∼0.80, not from 0.10 to 0.90. The thickness series maintains an equal thickness ratio of BTO to BFO with thicknesses close to the target values.

### 3.2. TEM

**Figure 3:**
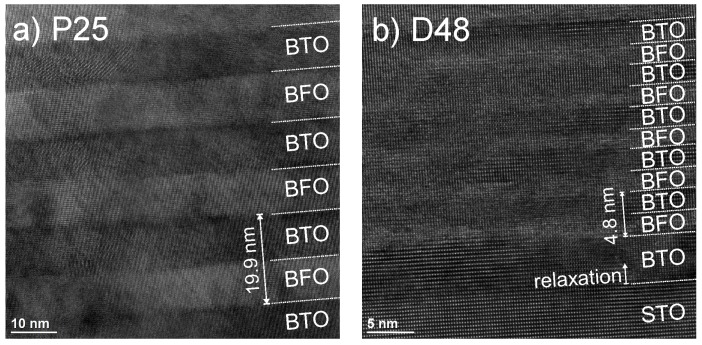
HR-TEM cross-sections of (**a**) P25/D200 middle segment (**b**) D48 near the substrate. Individual layers are marked accordingly, as well as the double layer thickness and crystallographic orientation.

We performed high-resolution transmission electron microscopy (HR-TEM) measurements on a selection of samples to supplement our findings on double layer thickness, individual layer thickness, coherency, crystalline quality, and lattice constants. Two of the resulting microscopy images are presented in Figure 3a sample P25 and Figure 3b sample D48) and the measurement results are summarized in Table 3. The overall $d_{\mathrm{dl}}$ values determined from superstructure fringe peaks in 2θ-ω scans, as well as the constituent layer thicknesses estimated by fitting XRR data match closely the values measured with TEM. Since this is the case for a representative cross-section of samples including the extremes of their respective series (highest, lowest, and standard BTO-BFO ratio, highest $p_{O_{2}}$, and highest and lowest $d_{\mathrm{dl}}$), we conclude that the XRD measurement results obtained for the remaining samples are also reliably accurate. In particular the sample P25 adheres to the target values for $d_{\mathrm{BTO}}$ and $d_{\mathrm{BFO}}$ closely, which makes for an ideal standard sample for our investigations. Both P25 and D48 display atomically smooth interfaces (see Figure 3). The sample R09 with the highest BTO content exhibits a significantly increased amount of strain contrast, as well as an interface roughness in the nm-range (see Figure S6a, which is consistent with the increased FWHM of this sample. While the sample R01 with the lowest BTO content displays atomically smooth interfaces, HR-TEM images from this sample feature many spots of Moiré patterns indicative of misaligned grains forming small angle grain boundaries within the film, as well as strain contrast, see Figure S6b.

The in-plane lattice constant of BTO changes from strained to the STO substrate to the average multilayer lattice constant within the first ∼5 monolayers for all samples, as indicated in Figure S5c. The constituent layers show no relaxation with respect to the distance from the interfaces and BTO and BFO can be said to be coherently strained to each other, as can be seen in Figure 3. All lattice constants measured by TEM are slightly larger compared to those determined by XRD measurements, but the same trends persist for the ratio and $d_{\mathrm{dl}}$ series: (a) $a_{\|\mathrm{ave}}\left(R09\right)>a_{\|\mathrm{ave}}\left(P25\right)>a_{\|\mathrm{ave}}\left(R01\right)$ and (b) $a_{\|\mathrm{ave}}\left(D48\right)≅a_{\|\mathrm{ave}}\left(P25\right)$. Overall the BTO lattice constants are consistent with a *c*-oriented tetragonal structure and the BTO lattice constants with a pseudo-cubic structure. Note that the in-plane lattice constants are derived from only one low-indexed peak each, which could explain the discrepancy of the absolute values between XRD and TEM, as low-indexed peaks are more prone to θ-dependent error.

### 3.3. Ferroelectric and Magnetic Measurements

**Figure 4:**
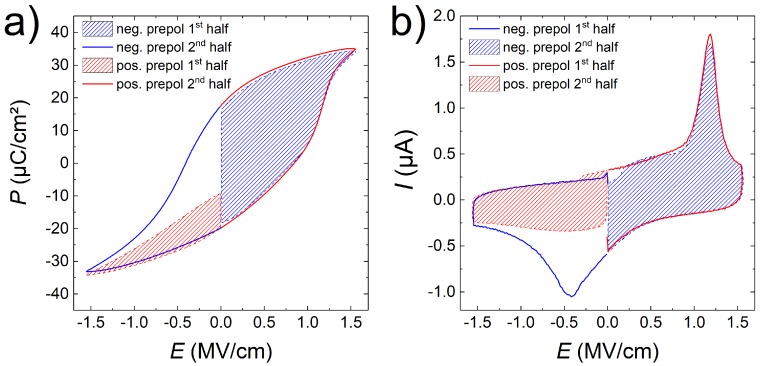
(**a**) P-E and respective (**b**) I-E curves recorded for sample D192 at a maximum voltage of 40 V. First (dashed lines, hatched) and second (solid lines) halves of the measurements performed after negative (blue) and positive (red) pre-poling, according to the measurement scheme presented in Figure S1.

All samples produce hysteresis curves in P-E measurements and show according ferroelectric switching peaks in the related I-E curves. Figure 4 shows representative ferroelectric measurements for the sample D192. As shown, the overall hysteresis is shifted to positive fields by on average ∼0.25 MV c$m^{-1}$, resulting in a self-poling effect towards the negative polarization state. This is illustrated in Figure 4b by the absence of a negative switching peak in the first half of the measurement following a positive pre-polarization pulse (red hatched area). The measurement procedure of the TF 2000 HS dynamic hysteresis measurement is shown in Figure S1. Since the first half of the measurement segment following the positive pre-polarization takes place after a delay time of 1 s, this is indicative that the polarization of our samples reverts to a mostly negative state in this time due to the imprint field. This is further evidenced by the appearance of the negative switching peak in Figure S7a P-E and Figure S7b I-E measurements at increasing maximum voltages) only after the voltage required to switch the polarization in the positive direction is reached. Typical saturation polarizations range from 20 to 30μC/cm${}^{2}$, comparable to values obtained for BTO-BFO bulk composites [20], but lower than some of our previous multilayers [13]. Overall many samples suffered from premature electrode breakdown at fields ≤1.2 MV c$m^{-1}$, which makes a systematic comparison difficult. We were recently able to drastically increase the breakdown fields of our samples to >2.0 MV c$m^{-1}$ through the reduction of droplet density and mosaicity by inserting a shadow mask during PLD (publication in preparation). The increase of the BTO content $d_{\mathrm{BFO}}/d_{\mathrm{dl}}$ from 0.3 to 0.8 leads to an increase of $P_{\max}$ from ∼20 to 30 μCc$m^{-2}$ at 40 V and a decrease of the imprint and coercive fields from 0.51 MV c$m^{-1}$ and 0.24 MV c$m^{-1}$ to 0.17 MV c$m^{-1}$ and 0.07 MV c$m^{-1}$.

All samples exhibit small ferromagnetic hystereses, an exemplary magnetic measurement is shown in Figure S8 for sample P25. The average saturation magnetization $M_{\mathrm{sat}}$ was 0.016 $\mu_{B}/\mathrm{formula}\;\mathrm{unit}$, with a remanent magnetization of $0.2\times M_{\mathrm{sat}}$ and a small coercive field of ∼15 mT.

### 3.4. Magnetoelectric Measurements

**Figure 5:**
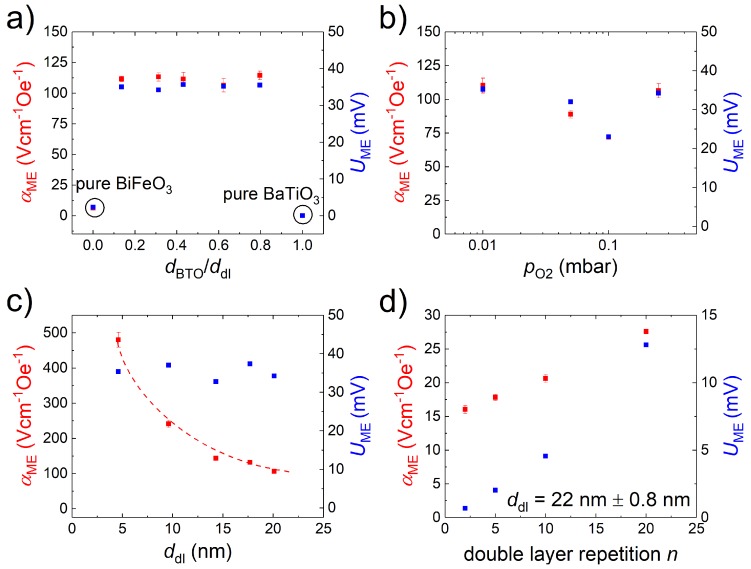
$\alpha_{\mathrm{ME}}$ values measured in 0 T at 300 K for the (**a**) BTO-BFO thickness ratio series; (**b**) $p_{O_{2}}$ series; (**c**) $d_{\mathrm{dl}}$ series; and (**d**) repetition *n* of double layers in a BTO-BFO multilayer stack (data adapted from [11]). The as-measured magnoelectric (ME) voltages are marked in blue, the $\alpha_{\mathrm{ME}}$ values in red.

In Figure 5a–c, we present the $\alpha_{\mathrm{ME}}$ values obtained for the above mentioned sample series in 0 T bias field at 300 K. Figure 5d additionally shows the relation of $\alpha_{\mathrm{ME}}$ on the double layer repetition *n* that was originally reported in [11]. If only the relative BTO and BFO thicknesses, not the overall $d_{\mathrm{dl}}$ is changed, a fairly constant $\alpha_{\mathrm{ME}}$ value is maintained for the multilayers (see Figure 5a. Depicted in Figure 5a are also the $\alpha_{\mathrm{ME}}$ values measured for ∼300 nm BTO and BFO single layer thin films with 0.01 Vcm${}^{-1}$Oe${}^{-1}$ and 6.42 Vcm${}^{-1}$Oe${}^{-1}$, as mentioned in Section 2.3.

As noted above, our more recent investigations suggested a certain influence of the double layer thickness, or alternatively of the BFO sublayer thickness of the multilayer stack on the magnitude of $\alpha_{\mathrm{ME}}$ [12,13]. This called into question the verifiability of the $p_{O_{2}}$ dependence of $\alpha_{\mathrm{ME}}$ previously reported in [8,9], where an invariant number of pulses led to a roughly exponential increase of $d_{\mathrm{dl}}$ with decreasing $p_{O_{2}}$. As Figure S9 shows, the dependency of $\alpha_{\mathrm{ME}}$ on log($p_{O_{2}}$) and on $d_{\mathrm{dl}}$ can be superimposed on one another fairly well. The $\alpha_{\mathrm{ME}}$ dependency on $p_{O_{2}}$ measured for the series of 16×(BTO-BFO) multilayers with a constant $d_{\mathrm{dl}}$ of 20±0.2 nm deposited in 0.01 to 0.25 mbar is shown in Figure 5b. While there is some variance of $\alpha_{\mathrm{ME}}$ with $p_{O_{2}}$, it is not monotonously decreasing with decreasing $p_{O_{2}}$, but rather exhibits a dip at 0.1 mbar and increases for lower and higher oxygen pressures. In fact the values measured for the 0.01 mbar and 0.25 mbar are almost identical at 110 Vcm${}^{-1}$Oe${}^{-1}$ and 106 Vcm${}^{-1}$Oe${}^{-1}$.

The explicit variation of $d_{\mathrm{dl}}$ with otherwise consistent deposition parameters and a 1:1 BTO-BFO thickness ratio leads a to roughly inverse dependency of $\alpha_{\mathrm{ME}}$ on $d_{\mathrm{dl}}$, as shown in Figure 5c. We reported similar dependencies based on the variation of the BFO layer only in [12,13]. For the thinnest sample D48, with each double layer consisting only of 6 unit cells each of BTO and BFO, an $\alpha_{\mathrm{ME}}$ value of 480 Vcm${}^{-1}$Oe${}^{-1}$ is obtained. This is the largest value recorded for any of our BTO-BFO multilayer samples and represents an increase of two orders of magnitude relative to the values reported for BFO single layers [13,19]. Note also that the ME voltage $U_{\mathrm{ME}}$ only slightly varies throughout the $d_{\mathrm{dl}}$ series.

An approximately linear relation of $\alpha_{\mathrm{ME}}$ on the repetition of double layers *n* is evident in Figure 5d. Noteworthy is that for small *n*, $\alpha_{\mathrm{ME}}$ tends toward a non-zero value, indicating a partial contribution of the ME coupling effect of the individual BFO layers.

**Figure 6:**
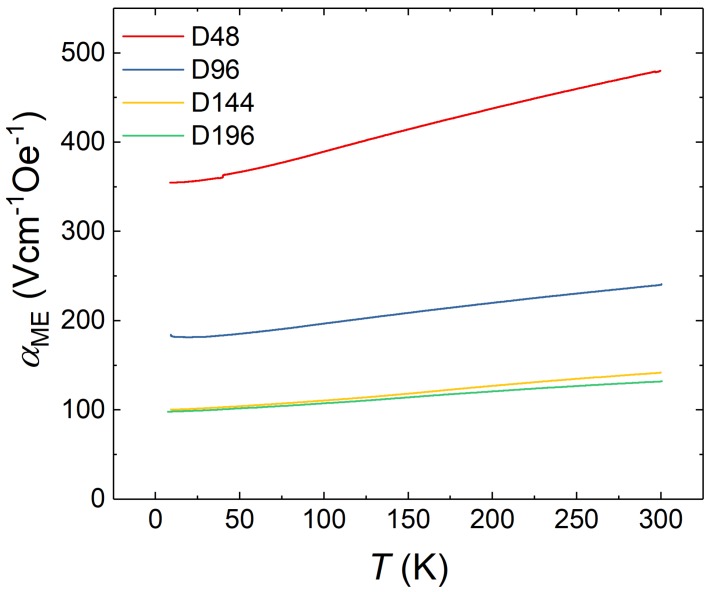
Temperature dependence of $\alpha_{\mathrm{ME}}$ measured in 0 T bias field for the samples of the $d_{\mathrm{dl}}$ series.

As presented in Figure 6, the temperature dependence of $\alpha_{\mathrm{ME}}$ is very similar for all samples of the $d_{\mathrm{dl}}$ series. Additionally, the temperature dependencies for the remaining samples can be found in Figure S10. For all samples investigated in this work, a monotonically rising behavior with increasing *T* persists.

**Figure 7:**
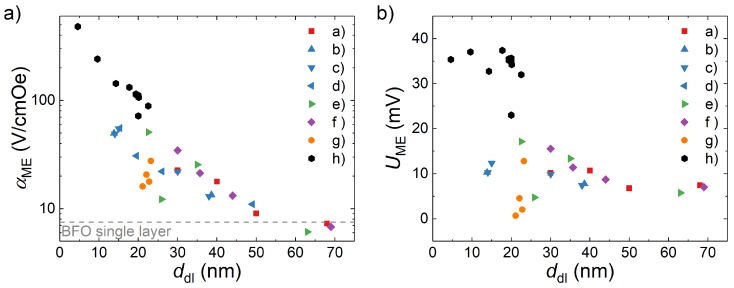
Overview: (**a**) $\alpha_{\mathrm{ME}}$ values and (**b**) respective as-measured $U_{\mathrm{ME}}$ values measured in 0 T at 300 K vs. $p_{O_{2}}$ for all BTO-BFO multilayers reported in a) [8] ($p_{O_{2}}$ variation); b)–d) [9,12,14] (BFO thickness variation); e) [13] (5% Gd substitution, BFO thickness variation); f) unpublished (see Table S1, BFO thickness variation); g) [11] (repetiton variation $n_{dl}=2–20$), and h) this work (BTO-BFO thickness ratio variation, $p_{O_{2}}$ variation, and *d*_dl_ variation).

Finally, in Figure 7 we present an overview of the $\alpha_{\mathrm{ME}}$ values for all BTO-BFO multilayer samples from our previous publications and this work, as well as some additional samples (BFO thickness variation, as listed in Table S1) in relation to their respective double layer thickness. Samples with $d_{\mathrm{dl}}$ over 60 nm show similar ME coupling values and monotonically falling *T*-dependency as BFO single layers. Multilayers with smaller $d_{\mathrm{dl}}$ appear to be limited by an approximately inverse exponential correlation of $\alpha_{\mathrm{ME}}$ to $d_{\mathrm{dl}}$. Note also, that the evolution of $U_{\mathrm{ME}}$ with $d_{\mathrm{dl}}$, shown in Figure 7b, is separated into two regimes. While for $d_{\mathrm{dl}}$ above 20 nm $U_{\mathrm{ME}}$ increases with lowered $d_{\mathrm{dl}}$, it caps at roughly 35 mV below 20 nm $d_{\mathrm{dl}}$. Hence the increase of $\alpha_{\mathrm{ME}}$ below this critical thickness is determined solely by the normalization of $U_{\mathrm{ME}}$ with $d_{\mathrm{dl}}$. While older samples, which were deposited with less optimized process parameters, fall behind in performance relative to newer sample series, they also follow a similar $d_{\mathrm{dl}}$ dependency trend within their own series.

## 4. Discussion

The fact that within the ratio series $\alpha_{\mathrm{ME}}$ only fluctuates by about 3 around the average value of 111 Vcm${}^{-1}$Oe${}^{-1}$ calls to question two previously held assumptions: That the enhanced ME coupling in BTO-BFO multilayers is mainly strain-mediated and depends highly on mosaicity, microstrain, and interface roughness. We have previously used the monotonically rising temperature dependency of $\alpha_{\mathrm{ME}}$ in thin multilayers to argue for strain-coupling as a potential source of the enhanced ME coupling [12], since the ferroelectric $d_{33}$ constant typically decreases with falling temperature for most perovskite ferroelectrics such as PZT [21] and BTO-BFO bulk composites [22]. However, from both theoretical predictions [15,16] and experiments on strain-coupled artificial multiferroic heterostructures [4,17] a strong dependence on the constituent material content would additionally be expected. The samples in this work had double layer thicknesses that varied by only <2%, while the FWHM of the superlattice (002) peaks changed by a factor of 5 from lowest to largest mosaicity (see Table 2). Neither this, nor the continuous strain tuning of $a_{\|\mathrm{ave}}$ with varied BTO content presented in Figure S5a or the change in ferroelectric saturation polarization appear to exert any significant influence on $\alpha_{\mathrm{ME}}$.

Similarly, a linear dependence of $\alpha_{\mathrm{ME}}$ on $p_{O_{2}}$ as previously reported in [8,9] is absent if $d_{\mathrm{dl}}$ is maintained relatively constant. The rise and fall of $\alpha_{\mathrm{ME}}$ with $p_{O_{2}}$ roughly correlates with $a_{\|\mathrm{ave}}$, as illustrated in the inset of Figure S5b, but not with the mosaicity. Hence the previously reported influence of $p_{O_{2}}$ dependent crystalline quality on $\alpha_{\mathrm{ME}}$ [8,9] may rather be connected to the double layer thickness.

The most consistent pattern of $\alpha_{\mathrm{ME}}$ enhancement can be found in the explicit $d_{\mathrm{dl}}$ and repetition series (see Figure 5c,d. Decreased distance between and increased number of interfaces in a multilayer stack both lead to an increase of $\alpha_{\mathrm{ME}}$. This is also consistent with the invariance of $\alpha_{\mathrm{ME}}$ with variation of BTO content with constant $d_{\mathrm{dl}}$. The same trend also persists as a limiting factor when considering the combined bulk of our work on BTO-BFO multilayers (see Figure 7). Furthermore, the as-measured, i.e., not thickness-normalized $U_{\mathrm{ME}}$ values saturate for $d_{\mathrm{dl}}$ below 20 nm. Similarly to the conclusion we previously drew from the change in temperature dependency [12], this points to two regimes of competing ME coupling mechanisms. With decreasing $d_{\mathrm{dl}}$ the coupling mechanism appears to change from resembling that of BFO single layers to one originating in close proximity to the BTO-BFO interfaces. As the separation length of the interfaces decreases, the contribution of the interface ME effect to the overall ME coupling increases down to a critical $d_{\mathrm{dl}}$ < 20 nm.

Thickness dependent cross-over of the dominant ME coupling mechanism is not entirely uncommon in literature. Examples are the strain and charge co-mediated coupling in Ni_0.79_Fe_0.21_/PMN-PT [23], (La,Sr)MnO_3_/BaTiO_3_ [24], and La_0.7_Sr_0.3_MnO_3_/PbZr_0.2_Ti_0.8_O_3_ [25] heterostructures. Charge-mediated ME coupling, however, typically produces much smaller $\alpha_{\mathrm{ME}}$ values, the same goes for other candidates of interface-driven effects such as interface orbital reconstruction [26,27] and charge ordering [26].

A potential source for a strong interface-driven magnetoelectric effect could lie in the coherent interfaces between BTO and BFO. The P4mm symmetric teragonal bulk BTO shows no oxygen octahedral tilt (OOT) with the polarization pointing along the $\left[001\right]$ axis, in contrast BFO with its rhombohedral R3*c* symmetry shows $a^{-}a^{-}a^{-}$ (in Glazer notation [28]) OOT and eight possible polarization directions along the ${\langle 111\rangle }_{p.c.}$ directions. The rotation of oxygen octahedra in perovskite oxides plays a crucial role in determining many of the materials’ properties, as especially magnetism and ferroelectricity are highly sensitive to variations in bond angles [29]. Disruption of OOT in heterostructures through geometric constraints at the interfaces can propagate over several nm from the interfaces [29,30,31]. Along with possibly compressive strain induced self-poling of the BTO and BFO layers [32,33], this interplay might also explain the large ferroelectric coercive and imprint fields, which could also contribute to the enhanced ME effect.

## 5. Conclusions

We have shown that by maintaining a consistent double layer thickness ($d_{\mathrm{dl}}$) in epitaxial BaTiO_3_-BiFeO_3_ multilayers, the effects of oxygen pressure, constituent layer thickness (down to ∼2 nm), interface roughness, and mosaicity on the magnetoelectric coupling coefficient $\alpha_{\mathrm{ME}}$ are miniscule, if not absent. The explicit variation of $d_{\mathrm{dl}}$ in multilayers with a 1:1 BaTiO_3_-BiFeO_3_ thickness ratio produces an explicit $\alpha_{\mathrm{ME}}$-$d_{\mathrm{dl}}$ dependency. This trend holds up when the collective results of our previous and current work on the topic is viewed through the same lens. The thinnest multilayer with a superlattice periodicity of only 4.8 nm ×16 produces a giant ME coefficient of 480 Vcm${}^{-1}$Oe${}^{-1}$, which signifies an enhancement of two orders of magnitude relative to BiFeO_3_ multilayers and is the largest we have measured for such a multilayer. The individual layers are relaxed with respect to the substrate and coherently strained to one another and show considerable ferroelectric coercive and imprint fields, as well as self-poling. The enhanced ME effect increases with temperature, but is otherwise not consistent with the widespread theory of the strain-coupled ME coupling theory applied to ferroelectric–ferromagnetic heterostructures. All signs point to an interface-driven origin of the enhanced ME coupling, though it would be conjecture to point at any specific origin at this point.

## Figures and Tables

**Table 1:** Samples used in this study. Naming schemes: R0*X*—nominal thickness ratio $0.X=d_{\mathrm{BTO}}/d_{\mathrm{dl}}$, P*YY*−$p_{O_{2}}=0.YY\;\mathrm{mbar}$, and D*ZZ*−$d_{\mathrm{dl}}^{\mathrm{nom}}=ZZZ\;\overset{˚}{A}$.

Sample Name	Series	$d_{\mathrm{BTO}}^{\mathrm{nom}}\left(\mathrm{nm}\right)$	$d_{\mathrm{BFO}}^{\mathrm{nom}}\left(\mathrm{nm}\right)$	$p_{O_{2}}\left(\mathrm{mbar}\right)$
R09	ratio	18	2	0.25
R07	ratio	14	6	0.25
R03	ratio	6	14	0.25
R01	ratio	2	18	0.25
P25	$p_{O_{2}}$	10	10	0.25
P10	$p_{O_{2}}$	10	10	0.10
P05	$p_{O_{2}}$	10	10	0.05
P01	$p_{O_{2}}$	10	10	0.01
D48	$d_{\mathrm{dl}}$	2.4	2.4	0.25
D96	$d_{\mathrm{dl}}$	4.8	4.8	0.25
D144	$d_{\mathrm{dl}}$	7.2	7.2	0.25
D192	$d_{\mathrm{dl}}$	9.6	9.6	0.25

**Table 2:** Results of XRD measurements. $d_{\mathrm{dl}}$ values are derived from superstructure fringes in 2θ-ω scans, $d_{\mathrm{BTO}}$ and $d_{\mathrm{BTO}}$ are derived from fits of XRR measurements, and $a_{\|\mathrm{ave}}$ values are derived from (103) RSMs. The error margin of $a_{\|\mathrm{ave}}$ is estimated to be ∼0.02 Å.

Sample Name	$d_{\mathrm{dl}}\left(\mathrm{nm}\right)$	${\mathrm{FWHM}}_{\mathrm{SL}001}{(}^{\circ })$	$d_{\mathrm{BTO}}\left(\mathrm{nm}\right)$	$d_{\mathrm{BTO}}\left(\mathrm{nm}\right)$	$a_{\|\mathrm{ave}}\left(\overset{˚}{A}\right)$
R09	19.4±0.6	0.066	15.7±0.8	3.8±0.2	4.00
R07	19.6±0.4	0.038	13.4±0.4	6.0±0.3	3.98
R03	19.4±0.6	0.123	6.0±0.1	13.3±0.4	3.97
R01	20.0±1.0	0.207	2.1±0.4	17.5±0.7	3.96
P25	20.1±0.6	0.085	8.5±0.5 ${}^{†}$	11±0.5 ${}^{†}$	3.97
P10	20.0±0.2	0.249	-	-	4.00
P05	22.5±0.7	0.062	11.4±0.4	11.3±0.6	3.99
P01	19.6±1.0	0.457	-	-	3.97
D48	4.6±0.2	0.048	-	-	3.97
D96	9.6±0.4	0.086	4.7±0.4	4.6±0.3	3.99
D144	14.3±0.2	0.088	7.2±0.1	7.1±0.1	3.97
D192	17.7±0.3	0.089	9.2±0.7	9.2±0.8	3.97

${}^{†}$ Values derived from TEM measurements.

**Table 3:** Results of TEM measurements.

Sample Name	$d_{\mathrm{dl}}\left(\mathrm{nm}\right)$	$d_{\mathrm{BTO}}\left(\mathrm{nm}\right)$	$d_{\mathrm{BTO}}\left(\mathrm{nm}\right)$	$a_{\|\mathrm{ave}}\left(\overset{˚}{A}\right)$	$c_{⊥\mathrm{BTO}}\left(\overset{˚}{A}\right)$	$a_{⊥\mathrm{BTO}}\left(\overset{˚}{A}\right)$
R09	19.4±0.5	15.3±0.5	3.9±0.4	4.08±0.02	4.06±0.02	4.11±0.03
R01	19.9±0.7	2.7±0.3	17.1±0.5	4.01±0.02	4.03±0.09	4.04±0.02
P25	19.9±0.2	9.5±0.5	10.3±0.5	4.04±0.04	4.15±0.05	3.97±0.16
D48	4.8±0.2	2.6±0.2	2.1±0.3	4.04±0.01	4.09±0.05	4.01±0.08

## Supplementary Materials

The following are available online at https://www.mdpi.com/1996-1944/13/1/197/s1, Figure S1: Measurement principle of the TF 2000 HS dynamic hysteresis measurement. (**a**) triangular voltage pulse sequence, (**b**) respective P-E loops, and (**c**) respective I-E loops. The polarization P is calculated by integration of the measured current I that results from the electric field E change and is normalized by the electrode area a, where a is determined by optical microscopy and E by division of the applied voltage V with the total film thickness. The final, true P-E loop consists of the second half of the two measurements performed after pre-polarization pulses leading to a negative (blue) and positive (red) pre-poled state. The solid lines represent the respective second halves of the measurements and start from a oppositely polarized stat. The first halves hence contain information about the polarization changes that take place in the 1 s delay time between pre-polarization pulse and measurement pulse, Figure S2: 2*q* − *w* scans for the samples of (**a**) the *p*_O_2__ series and (**b**) the BTO-BFO-ratio series, Figure S3: RSM around the STO 001 ((**a**–**d**)) and 103 ((**e**–**h**)) peaks for samples R09 ((**a**,**e**)), R07 ((**b**,**f**)), R03 ((**c**,**g**)), and R01 ((**d**,**i**), Figure S4: RSM around the STO 001 ((**a**–**d**)) and 103 ((**e**–**h**)) peaks for samples P25 ((**a**,**e**)), P10 ((**b**,**f**)), P05 ((**c**,**g**)), and P01 ((**d**,**i**), Figure S5: In-plane lattice constants derived from RSMs around the (103) STO substrate peaks for (**a**) the BTO-BFO ratio series, (**b**) the *p*_O_2__ series, and (**c**) the thickness series. The gray segmented lines in (**a**) mark the in-plane lattice constants of bulk STO (JCPDS 84-0444), BFO (pseudocubic, JCPDS 73-0548), and BTO (JCPDS 83-1880), as noted respectively, Figure S6: TEM images from samples (**a**) R09 and (**b**) R01, (**c**) in-plane lattice parameter evolution over the first 10 monolayers of sample D48, Figure S7: (**a**) P-E and (**b**) I-E loops recorded for sample D192 at voltages from 5 V to 40 V, Figure S8: VSM measurements for sample P25 performed at 10 K, 150K and 300 K. Figure S9. *a*ME plotted against ddl (black, lower scale) and *p*_O_2__ (red, upper log scale) for the BaTiO_3_-BiFeO_3_ multilayers reported in Lorenz et al. 2015. Figure S10: *a*ME plotted against T for (**a**) the *p*_O_2__ series and (**b**) the ddl series, Table S1: List of additional samples. ddl values derived from superstructure fringes in 2*q*-*w* scans, dBTO and dBTO derived from fits of XRR measurements.

## Abbreviations

The following abbreviations are used in this manuscript:

$\alpha_{\mathrm{ME}}$	magnetoelectric voltage coefficient
$d_{\mathrm{dl}}$	double layer thickness
ME	magnetoelectric
PLD	pulsed laser deposition
BTO	BaTiO_3_
BFO	BiFeO_3_
STO	SrTiO_3_
RSM	reciprocal space map
FWHM	full width at half maximum
TEM	Transmission electron microscope
HR-TEM	high-resolution transmission electron microscopy
PPMS	physical property measurement system
VSM	vibrating sample magnetometer
XRR	X-ray reflectometry
XRD	X-ray diffraction
OOT	oxygen octahedral tilt
