# Supplementary material for: Enhanced Magnetoelectric Coupling in BaTiO3-BiFeO3 Multilayers—An Interface Effect
Source: Materials (Basel). 2020 Jan 2;13(1):197. doi: 10.3390/ma13010197 (PMC6982203; doi:10.3390/ma13010197)
Supplement: Supplementary file 1 [file materials-13-00197-s001.pdf]

materials

MDPI

Article

Enhanced magnetoelectric coupling in $BaTiO_3$-$BiFeO_3$ multilayers - an interface effect

Stefan Hohenberger [1], Johanna K. Jochum [2,3], Margriet J. Van Bael [2], Kristiaan Temst [2], Christian Patzig[4], Thomas Höche[4], Marius Grundmann [1], and Michael Lorenz [1]

1 Felix-Bloch-Institut für Festkörperphysik, Universität Leipzig, Linnéstraße 5, D-04103 Leipzig, Germany
2 Laboratorium voor Vaste-Stoffysica en Magnetisme, Celestijnenlaan 200D, B-3001 Leuven, Belgium
3 Heinz Maier-Leibnitz Zentrum, Lichtenbergstr. 1, D-85747 Garching, Germany
4 Center for Applied Microstructure Diagnostics, Fraunhofer-Institut für Mikrostruktur von Werkstoffen und Systemen, Walter-Hülse-Straße 1, D-06120 Halle, Germany
* Correspondence: stefan.hohenberger@uni-leipzig.de

Received: 28 November 2019; Accepted: 23 December 2019; Published: 2 January 2020

check for updates

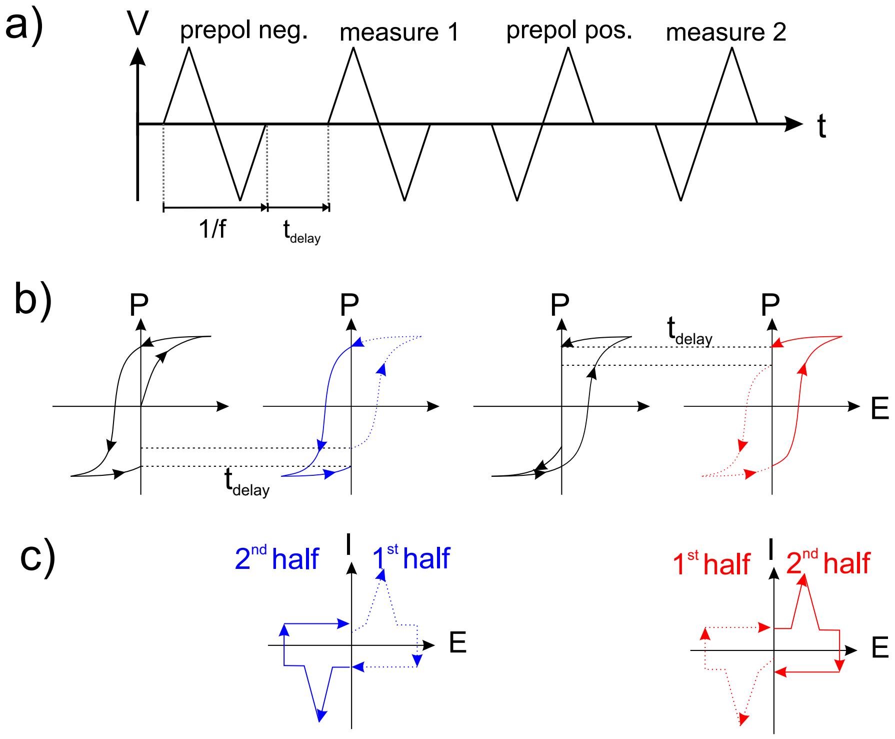

Figure 1. Measurement principle of the TF 2000 HS dynamic hysteresis measurement. (**a**) triangular voltage pulse sequence, (**b**) respective *P*-*E* loops, and (**c**) respective *I*-*E* loops. The polarization *P* is calculated by integration of the measured current *I* that results from the electric field *E* change and is normalized by the electrode area *a*, where *a* is determined by optical microscopy and *E* by division of the applied voltage *V* with the total film thickness. The final, true *P*-*E* loop consists of the second half of the two measurements performed after pre-polarization pulses leading to a negative (blue) and positive (red) pre-poled state. The solid lines represent the respective second halves of the measurements and start from a oppositely polarized stat. The first halves hence contain information about the polarization changes that take place in the 1 s delay time between pre-polarization pulse and measurement pulse.

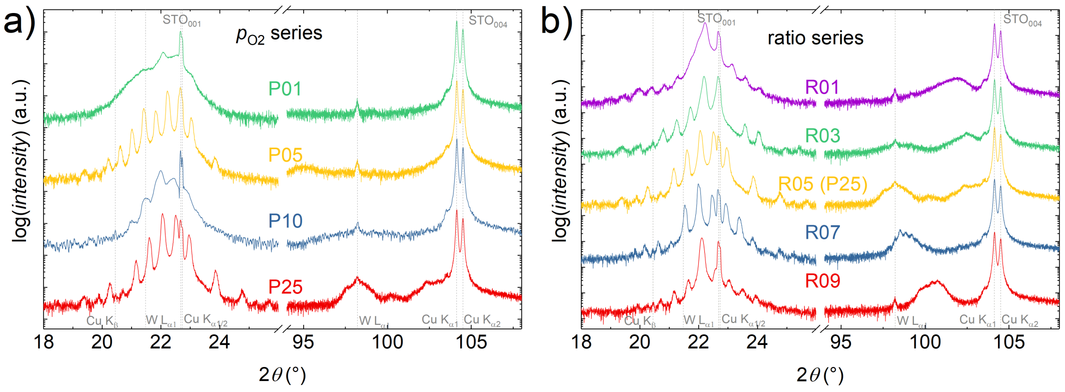

Figure 2. $2\theta - \omega$ scans for the samples of **(a)** the p_{O_2} series and **(b)** the BTO-BFO-ratio series.

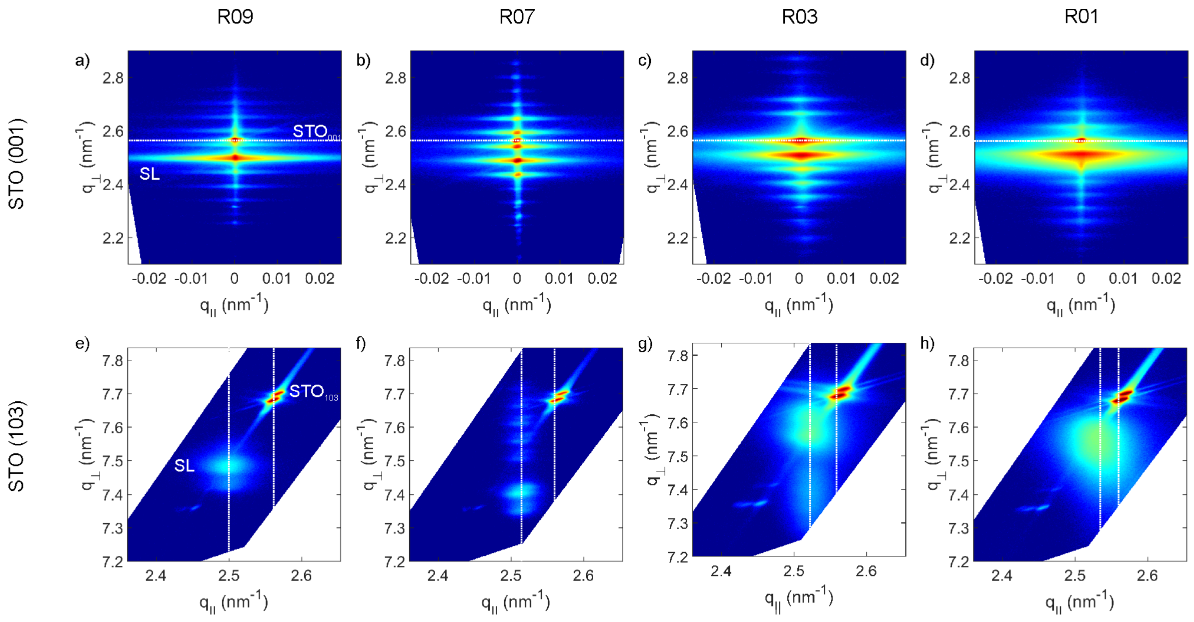

Figure 3. RSM around the STO 001 ((**a**)-(**d**)) and 103 ((**e**)-(**h**)) peaks for samples R09 ((**a**),(**e**)), R07 ((**b**),(**f**)), R03 ((**c**),(**g**)), and R01 ((**d**),(**i**).

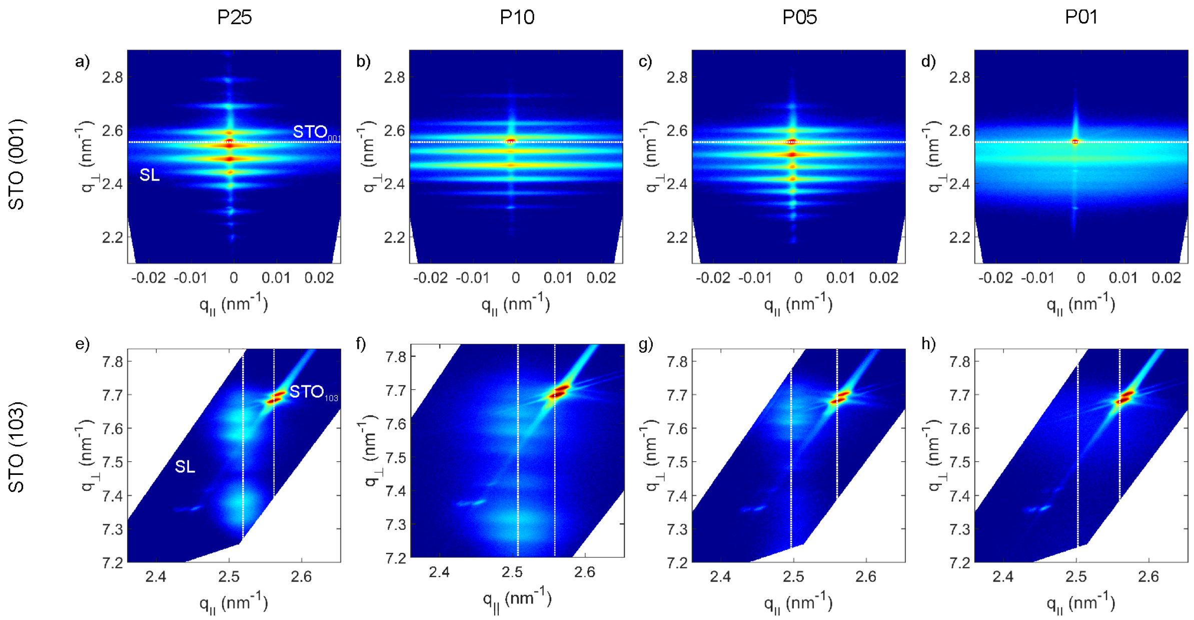

Figure 4. RSM around the STO 001 ((**a**)-(**d**)) and 103 ((**e**)-(**h**)) peaks for samples P25 ((**a**),(**e**)), P10 ((**b**),(**f**)), P05 ((**c**),(**g**)), and P01 ((**d**),(**i**))..

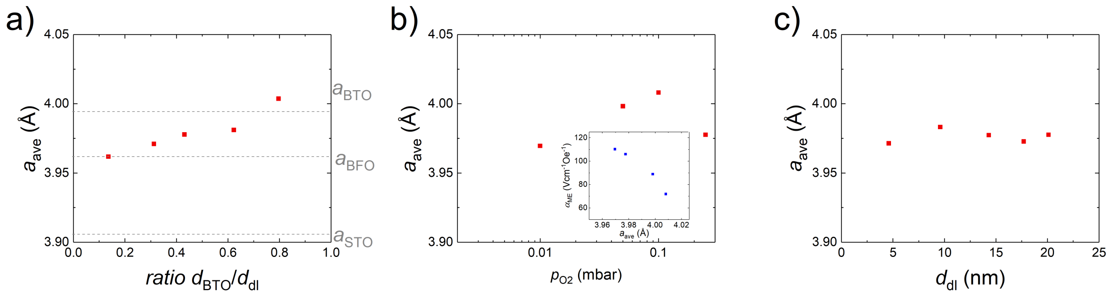

Figure 5. In-plane lattice constants derived from RSMs around the (103) STO substrate peaks for (**a**) the BTO-BFO ratio series, (**b**) the p_{O_2} series, and (**c**) the thickness series. The gray segmented lines in (**a**) mark the in-plane lattice constants of bulk STO (JCPDS 84-0444), BFO (pseudocubic, JCPDS 73-0548), and BTO (JCPDS 83-1880), as noted respectively.

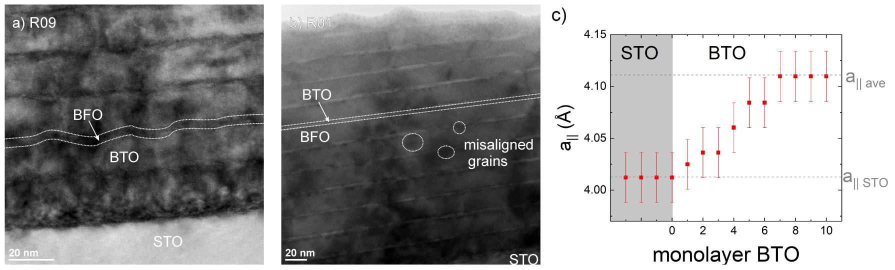

Figure 6. TEM images from samples (**a**) R09 and (**b**) R01, (**c**) in-plane lattice parameter evolution over the first 10 monolayers of sample D48.

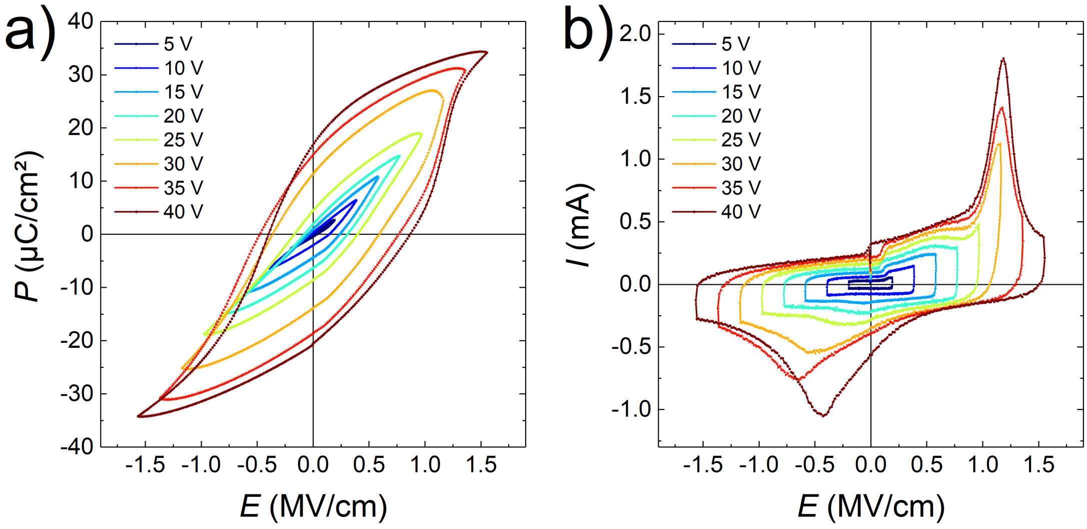

Figure 7. (**a**) *P*-*E* and **b**) *I*-*E* loops recorded for sample D192 at voltages from 5 V to 40 V.

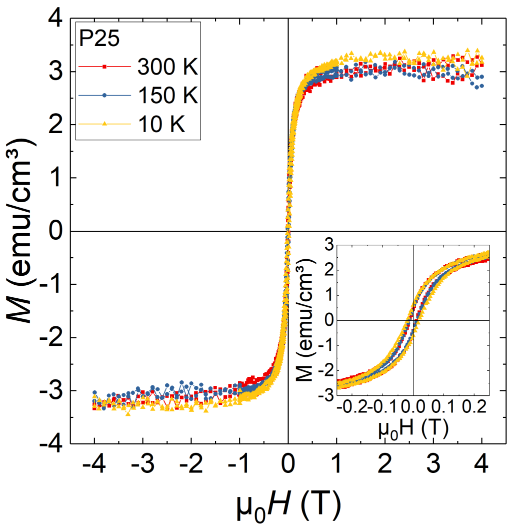

Figure 8. VSM measurements for sample P25 performed at 10 K, 150 K and 300 K.

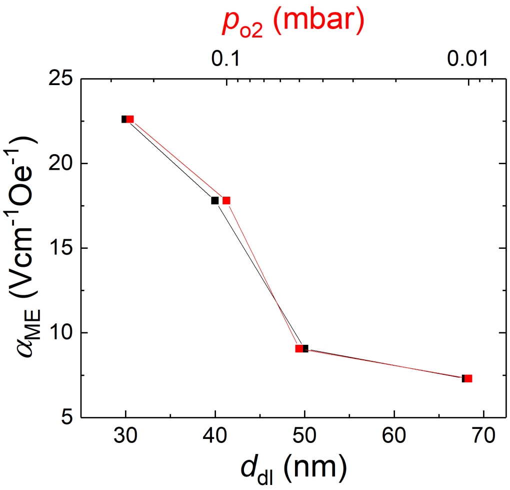

Figure 9. α_{ME} plotted against d_{dl} (black, lower scale) and p_{O_2} (red, upper log scale) for the $BaTiO_3$-$BiFeO_3$ multilayers reported in Lorenz *et al.* 2015.

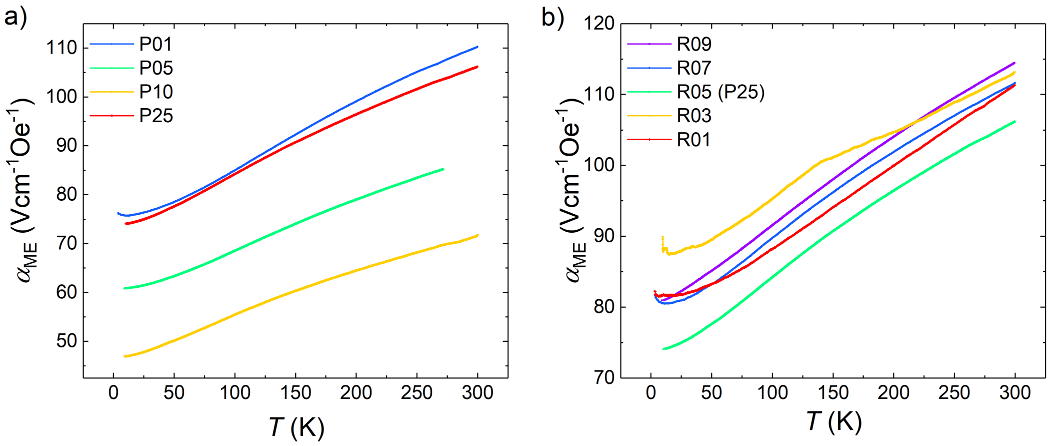

Figure 10. α_{ME} plotted against T for (a) the p_{O_2} series and (b) the d_{dl} series.

Table 1. List of additional samples. d_{dl} values derived from superstructure fringes in 2θ-ω scans, d_{BTO} and d_{BTO} derived from fits of XRR measurements.

sample name	d_{dl} (nm)	d_{BTO} (nm)	d_{BFO} (nm)
G6041	69.0 ± 4.0	$25.6 \pm 0.5^{\dagger}$	$43.6 \pm 0.5^{\dagger}$
G6043	44.0 ± 3.0	27.3 ± 1.3	16.3 ± 0.7
G6044	35.7 ± 0.8	27.6 ± 0.7	7.8 ± 0.3
G6045	30.0 ± 2.0	25.6 ± 1.2	3.5 ± 0.3

† Values derived from TEM measurements.

© 2020 by the authors. Licensee MDPI, Basel, Switzerland. This article is an open access article distributed under the terms and conditions of the Creative Commons Attribution (CC BY) license (http://creativecommons.org/licenses/by/4.0/).
